# Is Asphyxiating Thoracic Dystrophy (Jeune's Syndrome) Deadly and Should We Insist on Treating It? Reconstructive Surgery “On Demand”

**DOI:** 10.1055/s-0037-1598043

**Published:** 2017-02-17

**Authors:** Rosen Stanchev Drebov, Atanas Katsarov, Emiliyan Gagov, Nia Atanasova, Zlatin Penev, Alexander Iliev

**Affiliations:** 1 Department of Pediatric and Thoracic Surgery, University Multiprofile Hospital for Active Treatment and Emergency Medicine “Pirogov” Sofia, Bulgaria

**Keywords:** Jeune's syndrome, asphyxiating thoracic dystrophy, mandible locking plate, thoracic insufficiency syndrome

## Abstract

Our aim is to present the treatment of one of the skeletal manifestations of Jeune's syndrome (JS), the hypoplastic chest, which can result in thoracic insufficiency syndrome and present “on-demand” stage surgical technique using mandible locking plate system for the fixation of ribs. The diagnosis “Jeune's syndrome” was presented clinically in a 3-month-old girl from a family in which the first child died of JS at the age of 18 months. After close follow-up for several months and preoperative planning, we decided to make reconstructive chest operation with atypical use of a double-angled mandible locking plate for fixation. The plate was shaped as a “crown” to ensure the three dimension stability, from the dorsal part of the most curved ribs (paravertebrally) to the sternum after the resection of this area. Operation was done at the period of worsened breathing. For nearly 1 year, the rib cage preserved its stability and the child was in good condition. During the next 3 months, the upper part of the deformation started to grow inward fast. Second operation was “on demand,” and the implants used were mandible locking plates curved anterolaterally to effectuate extension of the rib cage and the sternum. In both the reconstructive operations, we spared the rectus and pectoral muscles and achieved good enlargement of the thoracic volume. The postoperative period is smooth and the child is active, without complications. We believe that in the future, the treatment should be “on demand” according to the course of the illness and the results of the follow-up examinations and adequate to the progress of chest wall deformity.

In 1955, Jeune et al described familial asphyxiating thoracic dystrophy in a pair of siblings with severely narrowed chests. 1 The term “asphyxiating thoracic dystrophy” has been used both as a synonym of Jeune's syndrome (JS) and as a diagnostic term for any instance of a severely constricted chest. 2

The gene responsible for JS ( *IFT80* ) is inherited in an autosomal recessive manner. 3 4 Most deaths are in the first 2 years of life due to respiratory failure after thoracic insufficiency syndrome (TIS). 5 The manifestations in skeletal system are dwarfism, hypoplastic chest (“bell” shape at anterior look) with short ribs, short limbs, polydactyly, and specific radiographic changes in the ribs and pelvis. 6 7 Some patients develop late ocular complications such as “retinitis pigmentosa.” 8

## Aim

To present the treatment of one of the skeletal manifestations of JS, the hypoplastic chest, which can result in TIS and present “on-demand” stage surgical technique using mandible locking plate system for the ribs' fixation.

## Case Report

**Fig. 1:**
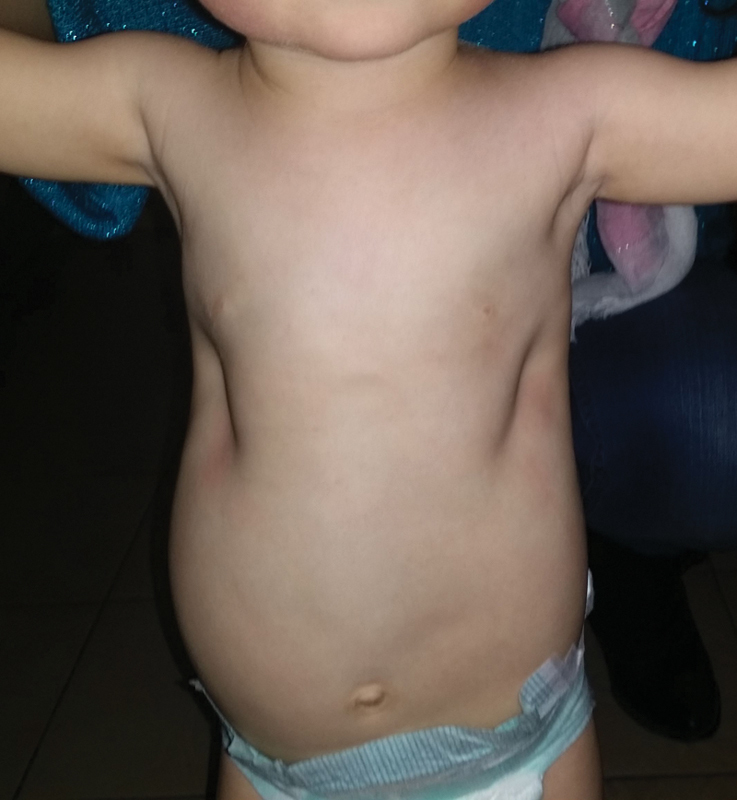
Front view of the child's chest before the first operation.

**Fig. 2:**
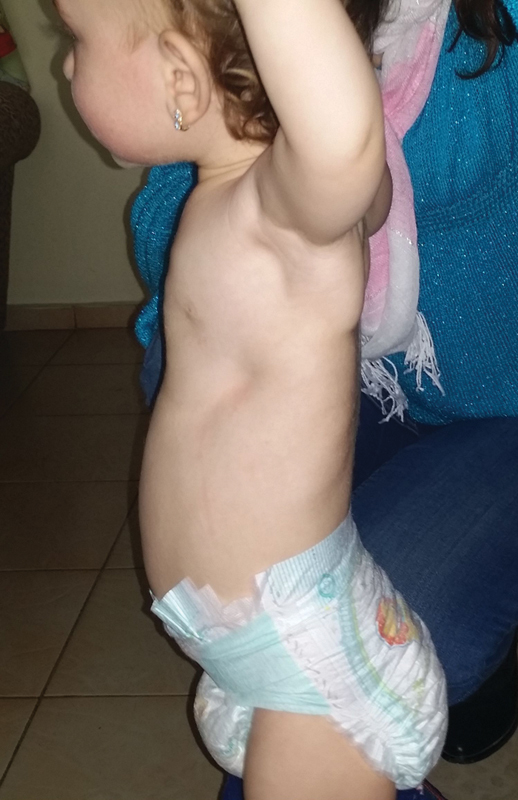
Left lateral view of the child's chest before the first operation.

**Fig. 3:**
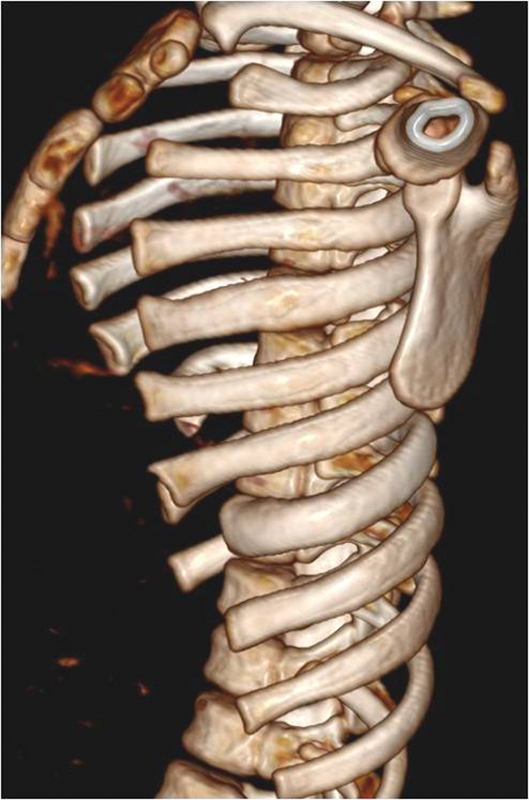
Three-dimensional reconstruction computed tomography scan of the chest 5 months before the operation; left lateral.

**Fig. 4:**
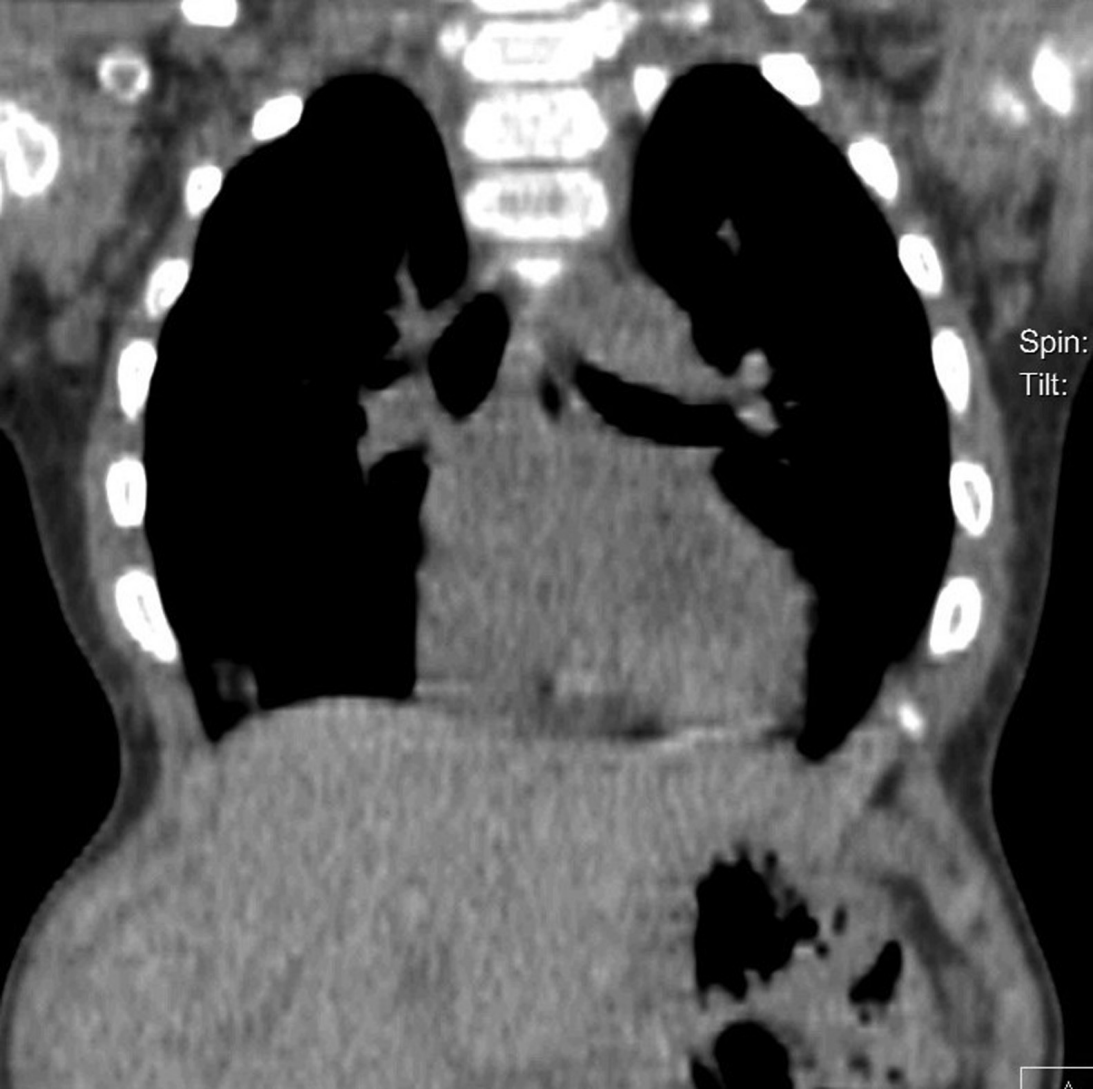
Computed tomography scan of the chest 5 months before the operation demonstrates the lung compression.

**Fig. 5:**
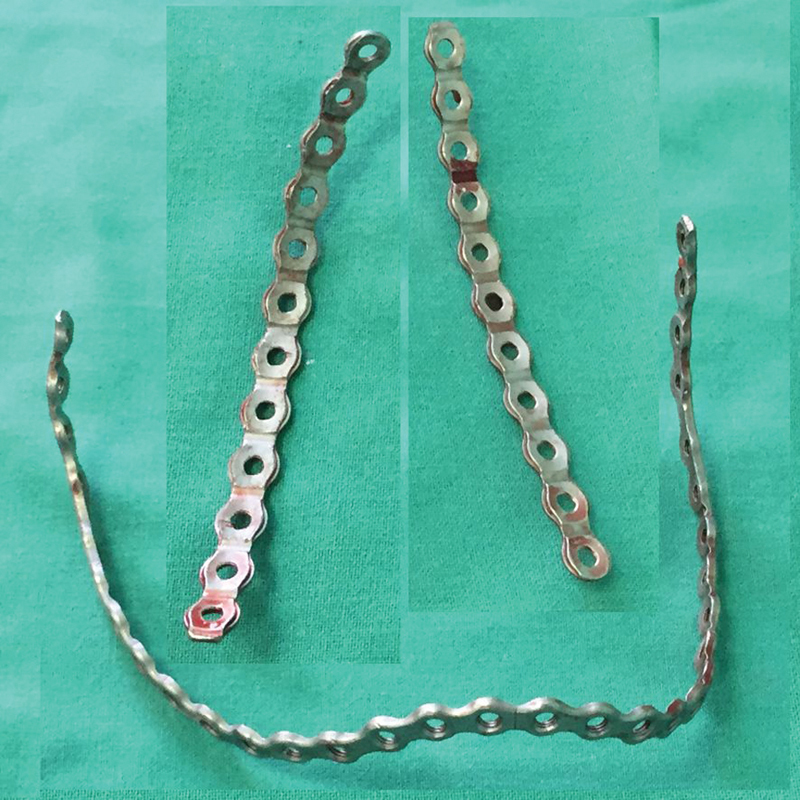
Contoured mandible locking plates used in the first operation.

**Fig. 6:**
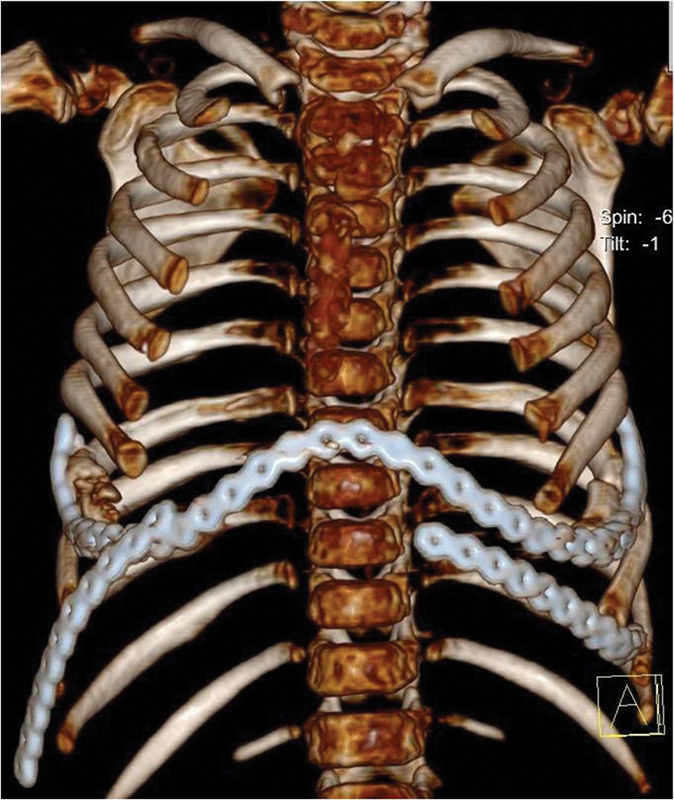
Three-dimensional reconstruction computed tomography scan of the chest after the first operation; anteroposterior view.

**Fig. 7:**
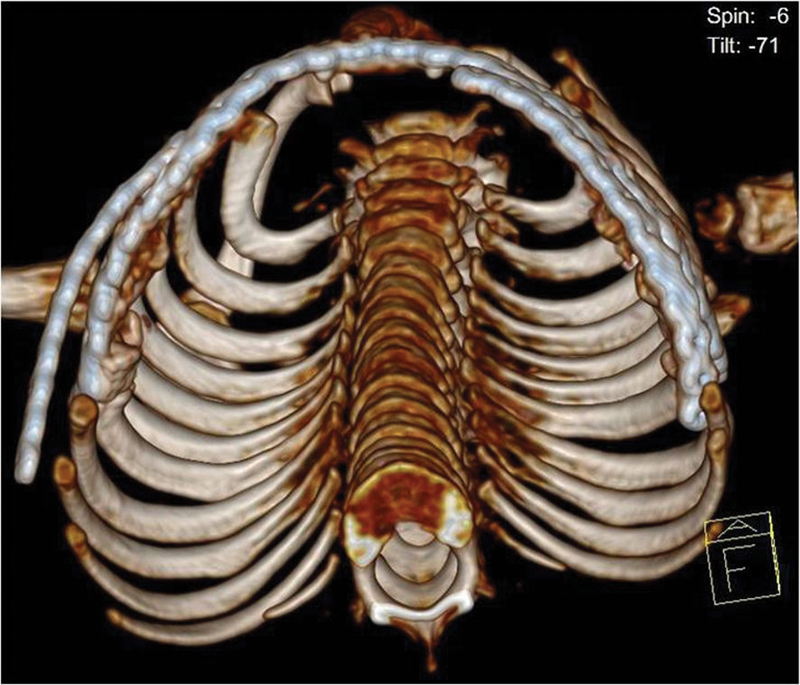
Three-dimensional reconstruction computed tomography scan of the chest after the first operation; axial view. Note the restored anterolateral contour of the chest.

The diagnosis “Jeune's syndrome” was presented clinically in a 3-month-old girl from a family in which the first child died of JS at the age of 18 months. Our little patient was with bilateral depression of the chest from the fourth to eighth rib, from the sternum to the anterior and midaxillary line ( Figs. 1 and 2 ). X-ray follow-up examination 3 months later showed progression of the deformity with the appearance of chest deformity and computed tomography (CT) image of lung compression ( Figs. 2 – 4 ). Due to logistic and financial problems, the operation was postponed for 5 months. In the end of that waiting period, the child had clinically presented TIS. First operation was indicated and done in that period of deterioration of respiratory function—main symptom of TIS. 9 It was proved by clinical findings and direct measurement of the lung volume at CT scan examination. It was 197.11 mL. We resected the deformed ribs (fifth to eighth) bilaterally and fix them with locking plate contoured in a “crown” shape and two shorter plates situated at both sides to sustain the resected ribs bilaterally ( Fig. 5 ). Postoperative X-rays showed excellent ribs position and good chest symmetry ( Figs. 6 and 7 ). The volume of the chest was increased (220.42 mL).

Five months later we performed implants removal operation. We realized that there was a risk of asymmetric chest growth because of the implants' rigidity.

For nearly 10 months, the rib cage preserved its stability and had clinically symmetric growth. The child was in a very good condition.

**Fig. 8:**
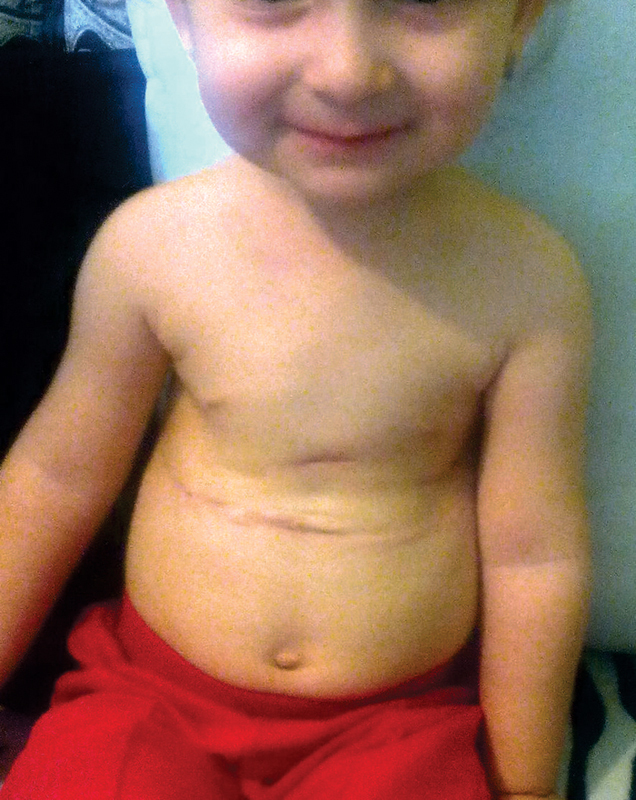
Front view of the child's chest before the second operation.

**Fig. 9:**
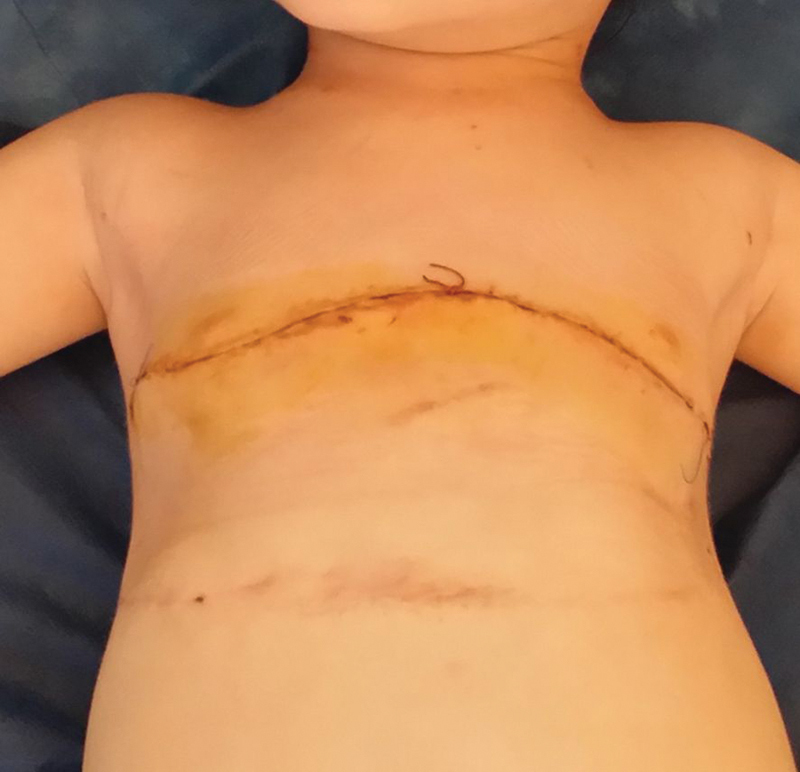
Front view of the child's chest immediately after the second operation (correction of the bell-shaped chest wall).

**Fig. 10:**
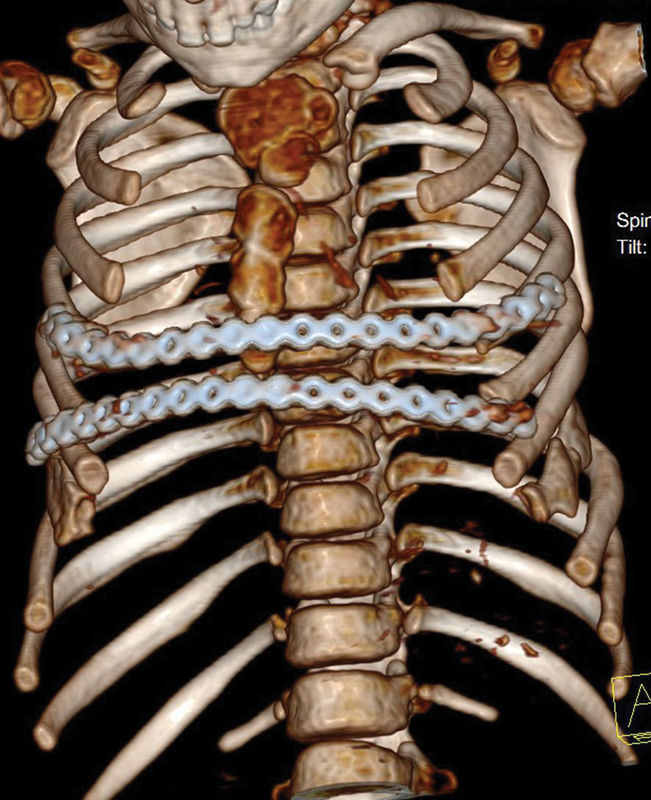
Three-dimensional reconstruction computed tomography scan of the chest after the second operation; anteroposterior view.

At the beginning of the 11th month after the first operation, we observed first clinical manifestation of a” bell-shaped” chest. During the next 3 months, the upper part of the previously nonfixed, but resected ribs started inward growing fast, forming the typically “bell” shape chest ( Fig. 8 ). We observed that this process started first from the costal cartilage and progressed to the bony parts of the ribs. The thorax was again asymmetrical and the value of thumb excursion test was +1. 10 That was the indication for our second operation. Through direct skin approach, we made chevron shape resections of the deformed ribs at two points ( Fig. 9 ). We used two short mandible locking plates placed anterolaterally for the ribs' fixation ( Fig. 10 ). Two plates sustain the chest volume and the resected ribs. The early postoperative period was without any complications. The direct lung volume measured was 232 mL.

**Fig. 11:**
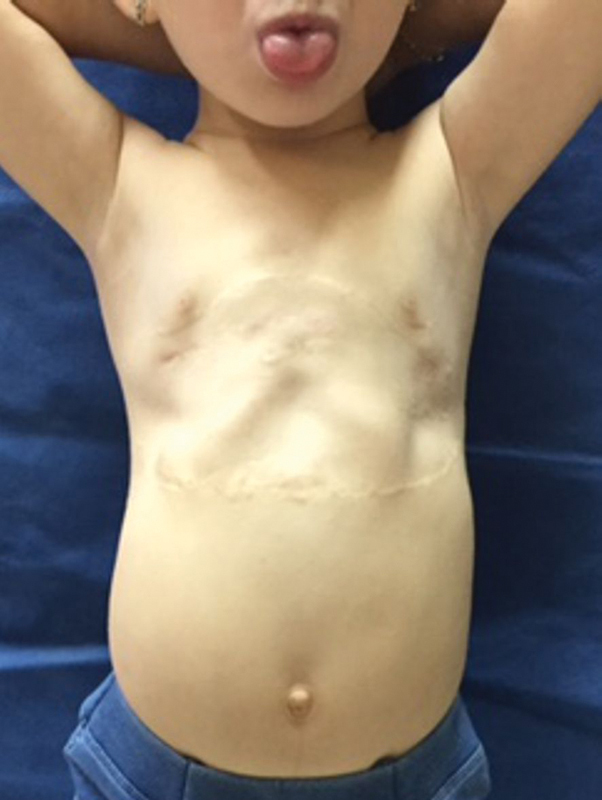
Front view of the child's chest 9 months after the second implant removal operation.

**Fig. 12:**
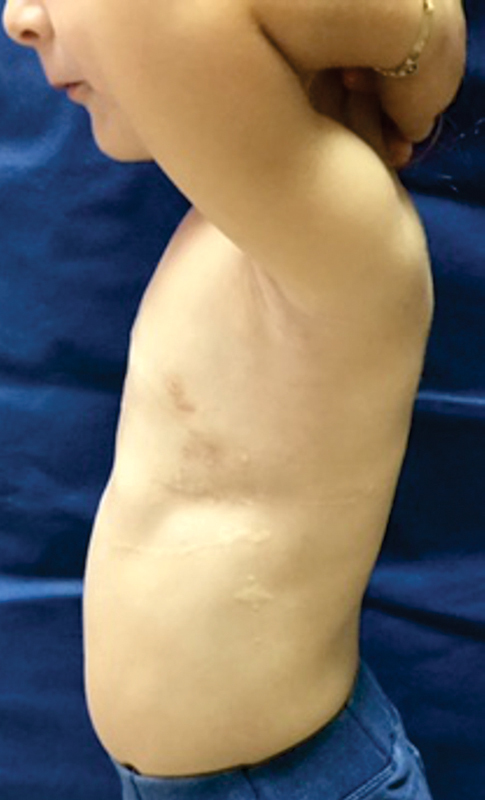
Lateral view of the child's chest 9 months after the second implant removal operation.

Three months later, the implants were removed. The appearance of the chest is symmetric vertically and horizontally ( Figs. 11 and 12 ).

## Methods

**Fig. 13:**
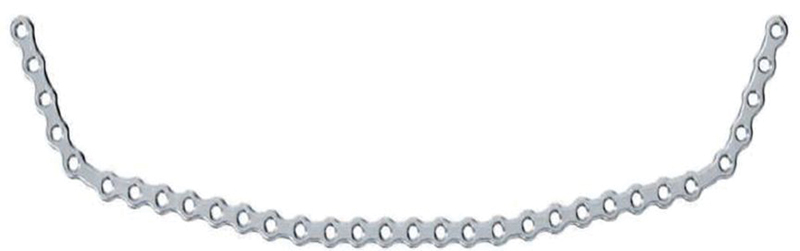
Long (32 holes) mandible reconstructive locking plate (scheme) used in the first operation.

The atypical use of a double-angled mandible locking plate was necessary because of the very small size and tenderness of the ribs ( Fig. 13 ). Our preoperative planning of the first operation was based especially on three-dimensional (3D) CT examination results and had to determine the specific intraoperative shaping of the implants and fixation points to the ribs. We evaluated the total chest hypoplasia by comparison of the circumference of the chest with normal value. We came to the conclusion that the shape of the implant must be similar to the rib contour of the lower part of the chest and resemble shape of a “crown.” We had to ensure the 3D stability, from the dorsal part of the chest, behind the most curved ribs, to the place over the rib resections. Plate was fixed by locking screws. We secured the plate fixation by absorbable sutures. The implant removal was done 5 months after the first operation. Second operative correction was done 9 months after the first. The need of it was estimated after clinical examination (thumb excursion test), X-ray, and CT examination. It was impossible to measure the respiratory capacity of the lungs (pulmonary function tests) because of the small age of the patient. 6 Loss of the chest wall mobility and chest asymmetry were main indications for second correction. We used two mandible locking plates to correct the deformity. During the operation, we resected four angulated ribs bilaterally, performing osteotomies and chondrotomies to achieve increase of the thoracic volume. The second implant removal was made 4 months after the second correction.

In both the reconstructive operations, we spared the rectus abdominis and pectoral muscles and inserted the plates under them immediately over the rib cage. Fixation was performed with small three-point locking screws and long-term absorbable sutures. We observed smooth postoperative period in all the operations.

## Results

The surgical decisions about patients with JS are very difficult and depend on the phenotype expression, the speed of evolution, and the time of clinical worsening. Main concept in surgical treatment of our patient was to enlarge the volume of the chest. We achieved it by the ribs' reconstruction after the first operation. Early postoperative result was good. Child had longitudinal growth of the thorax but not at width and depth. We observed that the rigidity of the implants constricted the chest additionally and removed the implants. In the next few months, the longitudinal chest growth was preserved but the deformity gradually relapsed. Basically, the “bell” shape of the chest prompt us for the second “on demand” operation. We cut four deformed ribs at two points around the rib angle and used two short mandible plates for fixation, sparing the rib's periosteum. We made good axial and frontal plane distractions of resected ribs with the mandible locking plates. Both the reconstructive operations were performed to enlarge the volume of the thorax. We took material for histological examination from different parts of the ribs. Histological examination of the material from costochondral junction showed spotty endochondral ossification with highly disorganized chondrocyte columns—a sign that some other authors found also. 11 12

## Discussion

Primary JS is a rare, specific multisystem genetic disorder with clinical manifestations in renal, digestive, respiratory, and skeletal systems, which lead to the death of half to three-fourths of patients, with incidence of 1 case per approximately 120,000 live births. 1 2 3 6 One of the most serious complications is the growth arrest of the chest at width and depth. It results to TIS, which is hardly diagnosed by pulmonary function tests in small age patients. 6 Diagnosis is based at clinical signs, clinical examination, loss of the chest wall mobility, chest asymmetry, and indices measured at radiographs and CT scans.

If it is proved that the deformity of the chest wall does exist but symptoms of JS are mild, normally there is no need to operate but must perform regular follow-ups. While there is indeed significant reduction of the chest volume since the patients themselves are small in stature, they may be able to live a relatively normal life in spite of the deformity.

Surgical treatment is according to the findings at any separate case, but the basic principle is to provide acute increase of the chest volume and sufficient place for the lungs.

Different authors suggested different decisions according to the specificity of the case.

Campbell et al proposed open-wedge thoracostomy or use of Vertical Expandable Prosthetic Titanium Rib (VEPTR) system in TIS cases. 9 13 We think that the use of VEPTR in our case would only maintain vertical extension but not the 3D increase of the chest volume. The main problem in our case is the growth deficiency of the chest at width and depth.

Some authors like Fette and Rokitansky used thoracoplasty with metal implants offering survival. 14

Aronson et al proposed homologous bone graft for expansion thoracoplasty. 15 Davis et al performed lateral thoracic expansion for the treatment of JS. 16 Some other authors made stage operations. Split of the sternum and gradually expand the divided sternum to a total of 3 cm widening, using a Leibinger midface distractor converting this technique of distraction osteogenesis leading to successful expansion of the ribs. 17

We believe that the treatment should be “on demand” according to the course of the illness and the results of the follow-up examinations and adequate to the progress of chest wall deformity.

## Conclusion

Surgical treatment of the chest deformity in JS is obligatory when TIS starts to develop. We think that conventional rib fixation plate cannot find place in treatment of patients with small dimensions of ribs and chest. That is why we recommend using such orthopaedic implants in similar cases concerning reconstructive operations of thorax.

No successful surgical techniques have been described in the literature for the treatment of TIS in childhood. 17 The future of our case is unknown. It depends on the evolution of the illness. Our strategy will be close observation of the patient with regular follow-ups and surgical decisions according to the development of the symptoms adapted to the individual requirements. This “on-demand surgery” must be performed before the development of TIS and any need of mechanical respiratory ventilation.
